# How Did the Media Report the Mining Industry’s Initial Response to COVID-19 in Inuit Nunangat? A Newspaper Review

**DOI:** 10.3390/ijerph182111266

**Published:** 2021-10-27

**Authors:** Matthew Pike, Ashlee Cunsolo, Amreen Babujee, Andrew Papadopoulos, Sherilee L. Harper

**Affiliations:** 1 Department of Population Medicine, University of Guelph, Guelph, ON N1G 2W1, Canada; apapadop@uoguelph.ca; 2 School of Arctic and Subarctic Studies, Labrador Institute of Memorial University, Happy Valley-Goose Bay, NL A0P 1E0, Canada; ashlee.cunsolo@mun.ca; 3 School of Public Health, University of Alberta, Edmonton, AB T6G 2R3, Canada; babujee@ualberta.ca (A.B.); sherilee.harper@ualberta.ca (S.L.H.)

**Keywords:** Inuit, indigenous, arctic, health, well-being, COVID-19, pandemic, resource extraction, mining, media

## Abstract

Mining in Inuit Nunangat relies on a southern Canada fly-in/fly-out (FIFO) and local workforce. The FIFO workforce, combined with existing social determinants of health, can create health risks to Inuit Nunangat. These risks were increased with COVID-19. As newspaper reporting can shape public opinion and policy actions regarding these COVID-19 risks, we systematically searched databases to identify newspaper articles during the initial phase of COVID-19 (i.e., articles published from 1 January to 30 June 2020). Descriptive statistics and qualitative thematic analysis were used to analyze the nature, range, and extent of included articles. Most included articles were published by Inuit Nunangat-based newspapers. Half the sources quoted were mining companies and most reported reactions to their initial response were negative. The most frequent topic was concern that an infected FIFO employee could transmit COVID-19 to a worksite and subsequently infect Inuit employees and communities. Inuit Nunangat-based newspapers were crucial in shaping the narrative of the initial response. National newspapers mainly focused on the takeover of TMAC™ during the pandemic, while Inuit Nunangat-based newspapers provided timely and locally-relevant pandemic information. Without Inuit Nunangat-based newspapers, the reporting would be from national and southern newspapers, which was less in-depth, less frequent, and less relevant to Inuit.

## 1. Introduction

Indigenous Peoples have been the natural stewards of their traditional lands since time immemorial, relying on the lands and waters for sustenance, livelihoods, cultural connections, ceremony, and wellbeing [1]. Yet, since the advent of the ‘Age of Exploration’ [2], and processes of global colonization, Indigenous lands and waters have been the site of often-forced large-scale resource development and extraction projects, including oil and gas development [3], hydroelectric projects [4], deforestation [5], industrial development [4], and mining [5]. 

Over recent decades, extractive industries globally, such as mining, have been recognized as the “greatest threat” to Indigenous Peoples, as their traditional lands are often confiscated, leading to impacts on Indigenous cultures, health, food sovereignty and languages [1]. In many cases, mining operations rely heavily on fly-in/fly-out (FIFO) workers, leading to an influx of people from diverse backgrounds and geographies entering into Indigenous lands, and often having contact with the local workforce and populations [6]. In recent decades, with mining operations expanding deeper into previously untouched (by outside actors) and predominantly Indigenous regions, FIFO has become a preferred method of mining employment by Industry and Governments, mainly due to economic and logistical reasons [6,7,8].

However, this FIFO work structure has led to a number of social issues and concerns, for employees and surrounding populations, including: increased drug and alcohol usage [6]; increased rates of physical and sexual violence [6,7]; increased family stress; negative mental health impacts (e.g., depression, anxiety, suicide ideation) [8]; and decreased physical health [8]. Further, the increase in FIFO workers can also introduce infectious diseases to regions [6,9], increasing the health risks of Indigenous Peoples located near mining operations around the world—a health challenge which has emerged most acutely during the COVID-19 global pandemic [10]. 

For example, in Canada, there are significant mining operations on Indigenous lands, including in Northern Canada and Inuit Nunangat (Inuit Homelands) [11,12], where the remote location (in comparison to southern Canada) of these mines relies almost exclusively on a FIFO workforce, containing a mix of local and transient workers from southern Canada [10]. For mining companies operating in Inuit Nunangat, where many health challenges and lack of access to health-sustaining resources are well documented [13], the risk of transmissible diseases entering into local communities has been both a concern and a possibility; but, with the advent of the COVID-19 global pandemic, this concern became very real in early 2020 [10]. With a mix of Inuit Nunangat-based employees and southern Canada-based employees flying in and out [14], close living and dining quarters [15], minimal on-site medical facilities [15] and the high transmission rate of COVID-19 [16], mining operations in Inuit Nunangat presented a scenario which the World Health Organization (WHO) were warning mining companies to avoid [16]. The WHO also stated, through the media in early March 2020, that despite their mandate being public health, they were also cognizant of the social and economic impacts, and that every sector and individual would be impacted by the pandemic [16]. 

By early March 2020, mining companies in Inuit Nunangat were confronted with the realities of their Inuit employees and their communities: lack of access to adequate healthcare, overcrowding and shortage of quality and safe housing, significant underlying physical and mental health challenges, capacity issues in dealing with a pandemic, and a difficult history with pandemics [17,18]. These social determinants of health, among others, are deeply rooted in colonialism [19], and Indigenous Peoples, including Inuit [20], continue to face health care systems entrenched with racism [21]. Mining companies in Inuit Nunangat were faced with difficult decisions to continue operating and providing economic support [22] or shut down to prevent the virus from getting to their remote, Northern mine sites, and subsequently into vulnerable, predominantly Inuit communities [10].

In the early months of the pandemic, there was a heavy reliance on the media for COVID-19 related information as the developing situation led to continuous updates [23] [24]. Media, including newspapers, are essential tools in communicating what public health risks are, how serious they are and what can be done about them. The public, especially when assessing risk, often rely on media when formulating perceptions, opinions, and reactions to an event or topic. Furthermore, newspapers have the ability to frame a topic [25], which influences how the topic is viewed from a valence (positive, negative or neutral) perspective [26]. Mining companies, with a combined FIFO workforce numbering in the thousands from southern Canada, urgently needed to understand the impact to Inuit mining employees and their communities, and react to the heightened risk of COVID-19 transmitting to their mine sites via southern Canada FIFO workers.

A newspaper review in this context is helpful, given few studies that have explored the media’s treatment of Arctic issues [27]. Furthermore, the media’s treatment of Inuit-specific issues [28], and Indigenous issues in Canada overall [29,30], have been challenged as inadequate and inappropriate. As such, the research goal of this paper was to characterize the nature, range, and extent of national and regional newspaper coverage in Canada of the mining industry’s initial response to the COVID-19 pandemic in Inuit Nunangat. Specifically, this paper seeks to answer the question: How did the media report the mining industry’s initial response to the COVID-19 pandemic in Inuit Nunangat? 

While this paper focuses on the media’s reporting of the mining industry’s initial response to COVID-19 in Inuit Nunangat, this study can be applicable to future research of understanding the media’s reporting of other crises in Inuit Nunangat [27] and how the Government or Industry proponent response was covered by the media. Additionally, this research can be helpful to understand more broadly how regional and national media report on crisis events and/or the mining industry in Inuit Nunangat (for example, locally based reporting versus parachute reporting [31]) and other Indigenous communities both in Canada [29] and globally. Additionally, it could be helpful in understanding how media reporting impacts decision making and actions of Industry proponents when faced with external pressures.

## 2. Materials and Methods

### 2.1. Inuit Nunangat

Inuit Nunangat is the traditional homelands of the approximately 65,000 Inuit in Canada, who are represented nationally by Inuit Tapiriit Kanatami (ITK) [32]. While only representing 0.002% of the total Canadian population [33], Inuit Nunangat spreads across 51 communities in the Arctic and Subarctic regions of Canada, encompassing approximately 35% of Canada’s land mass and 50% of the country’s coastlines [32]. Inuit and their ancestors have occupied the Circumpolar North for thousands of years and maintain a rich Inuit culture, including hunting, trapping, fishing, and gathering [34]. Most Inuit communities have gone through significant and rapid transformation in recent decades [35], with many being impacted by forced resettlements [36] and resource development [37]. Additionally, climate change has resulted in important environmental changes that have directly and indirectly impacted Inuit [38], and is making Inuit Nunangat more accessible to extractive industries as the climate warms [39]. 

**Figure 1:**
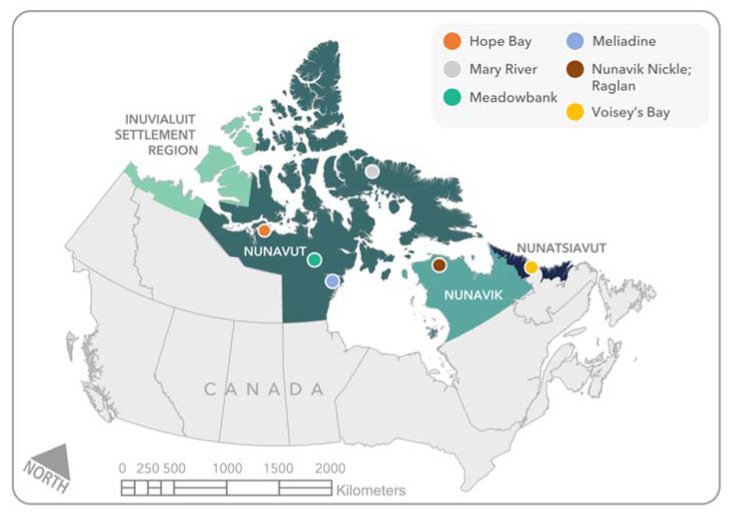
Map of operating mines in Inuit Nunangat, as of 30 June 2020.

Inuit Nunangat is also home to significant mineral reserves [40,41], along with several operating mines (Figure 1), including TMAC’s™ Hope Bay gold mine (On 5 January 2021, Agnico Eagle^®^ reached an agreement to purchase TMAC™, after the Government of Canada rejected the proposed plan for SD Gold to purchase TMAC™: Accessed on 9 January 2021: https://www.theglobeandmail.com/business/industry-news/energy-and-resources/article-agnico-eagle-mines-to-buy-TMAC-resources-after-bid-from-chinas/) (Nunavut—in operation since 2017), Agnico Eagle’s^®^ Meadowbank (Nunavut—in operation since 2010) and Meliadine (Nunavut—in operation since 2019) gold mines, Baffinland’s^®^ Mary River iron ore mine (Nunavut—in operation since 2015), Glencore’s^®^ Raglan nickel/copper mine (Nunavik—in operation since 1997), Canadian Royalties’^®^ Nunavik Nickel Project nickel/copper mine (Nunavik—in operation since 2013) and Vale’s^®^ Voisey’s Bay Nickel/Copper/Cobalt (Nunatsiavut—in operation since 2005) mine. These mines often provide some of the highest paying jobs in Inuit Nunangat, making them an attractive location to work for some Inuit [42]. For example, Agnico Eagle’s^®^ gold mining operations in Nunavut employed nearly 400 Nunavummiut in 2019, leading to what the company says is the growth of “an almost non-existent middle class” in Nunavut [43]. However, the significant concern expressed by Inuit on the negative impacts of a mine closure on Inuit and their communities is well documented [22], as with more reliance on mining and an increase in jobs, means the increase in negative impacts once the mineral resource is fully mined and the jobs are no longer available [22]. 

With the onset of the COVID-19 pandemic, a permanent or temporary mine closure was a high possibility in Inuit Nunangat. However, protecting the health and wellbeing of Inuit communities was the top priority, with ITK playing a lead role in ensuring Inuit were considered and supported as the pandemic reached Canada and Inuit Nunangat [44]. On 12 March 2020, a day after the WHO declared COVID-19 a pandemic, ITK President Natan Obed called on the Government of Canada to consider Inuit a high-risk group for any federal funding considerations provided to attempt to lessen the impacts on Inuit [44], who face long-standing social and economic inequities [45]. A steadfast focus on preventing the transmission of the virus to Inuit Nunangat emerged [46], with mining companies quickly becoming a focus for Inuit, Government and Public Health officials, given the significant FIFO workforce [47,48,49].

### 2.2. Theoretical Framework

This review used a comprehensive and systematic approach to identify relevant newspaper articles about the mining industry’s initial response to COVID-19 in Inuit Nunangat. In this review, the analysis is framed by the understanding that the media has an influential role on what the public should “think about, know about, have feelings about [50]” through agenda setting [50,51,52,53]. Studies have shown that media coverage significantly affects the salience, and therefore public awareness and agenda setting, of an issue [50,51]. Quantity of media coverage (i.e., number of newspaper articles), for example, is argued to be one of the most important factors in generating public reaction and opinion [51,52]. Furthermore, via terminology, tone, and themes, the media holds power in framing issues, therefore significantly influencing how the public understands and perceives an issue [27,51,53]. In this study, we examined how national and regional newspapers framed the mining industry’s initial response to COVID-19 in Inuit Nunangat. In particular, we focused on how newspapers framed the issue through valence, sources quoted, quantity of articles and other article characteristics.

### 2.3. Search Strategy

A systematic approach to reviewing newspaper coverage was used to identify English and French articles about COVID-19 and the mining industry in Inuit Nunangat, published between 1 January 2020, and 30 June 2020. This timeframe captured newspaper articles published prior to WHO declaring COVID-19 a pandemic on 11 March 2020, and encompasses the ‘first wave’ of COVID-19 in Canada and the ‘initial response’ to COVID-19 in Inuit Nunangat. Based on consultations with a University of Guelph librarian, a search string consisting of geographic, COVID-19, and mining terms (Table 1) was developed and used to search the ProQuest^®^ database (ProQuest LLC, Ann Arbor, MI, USA). The ProQuest^®^ database was selected as it included a significant number of Canadian national and regional newspapers; however, it did not include several locally important newspapers. Therefore, the following locally important newspapers were also searched, utilizing a modified search (Table 1) process: Nunatsiaq News, NNSL Media, and Labrador Voice. These additional newspapers were important to include as they were located in and provided coverage of the different regions of Inuit Nunangat where there were operational mines. The initial test searches were conducted on 1 June 2020, and the final search was conducted on 3 July 2020. Search results were uploaded to Mendeley^®^ for de-duplication. The remaining citations were then uploaded onto DistillerSR^®^ (Evidence Partners, Ottawa, ON, Canada) for additional de-duplication and to facilitate screening.

### 2.4. Inclusion and Exclusion Criteria

Inclusion and exclusion criteria were developed *a priori* for the two-stage screening process that was conducted by two independent reviewers. To be included, articles had to mention an operational mine in Inuit Nunangat within the context of COVID-19 (Table 2). For the purpose of this review, only operational mines located in Nunavik, Nunavut, and/or Nunatsiavut were included (there were no operational mines in Inuvialuit during the study period). Articles were excluded if they focused on non-operational mines or a mine located outside of one of the four settled Inuit land claim regions in Canada. Articles had to connect the mines to COVID-19 in some capacity (e.g., cases in workers, operational changes). Articles that discussed operational mines in Inuit Nunangat and COVID-19, but did not link the two, were excluded. Two independent reviewers used the inclusion and exclusion criteria to examine the full texts of each news article for relevance. The reviewers met throughout the screening process to discuss and reconcile any disagreements. The level of agreement between reviewers was calculated.

### 2.5. Data Extraction and Analysis

Articles that met the inclusion criteria underwent data extraction and analysis. Analysis focused on capturing how the media reported the mining industry’s initial response to the COVID-19 pandemic in Inuit Nunangat. First, a data extraction form with concepts developed *a priori*, along with data-driven concepts, was utilized to capture characteristics, such as: article attributes (e.g., newspaper outlet, publication date); mine characteristics (e.g., location, mining company, minerals extracted); sources quoted (e.g., company spokesperson, Inuit community member); COVID-19 related issues (e.g., reactions to industry response, impact on mine operations); and framing, including valence statements (valence regarding the mining companies’ initial response to COVID-19 is assessed. For example, if an article discussing a mining company’s response to COVID-19 elicited anger or praise from Inuit community members, it was considered a negative or positive valence statement, respectively. If an article contained both positive and negative valence statements, it was classified as neutral). To facilitate this extraction, Microsoft^®^ Excel (Microsoft, Redmond, WA, USA) was used to create a form and manually extract data from each relevant article (Table 3). Next, a hybrid process of deductive and inductive qualitative thematic analysis was used to interpret and analyze the relevant articles [54], which included the following phases: reviewing the data (i.e., article text); generating initial codes; searching for themes; reviewing themes; and defining and naming themes [55].

## 3. Results

### 3.1. Database Search Results and General Article Attributes

#### 3.1.1. Database Search Results

**Figure 2:**
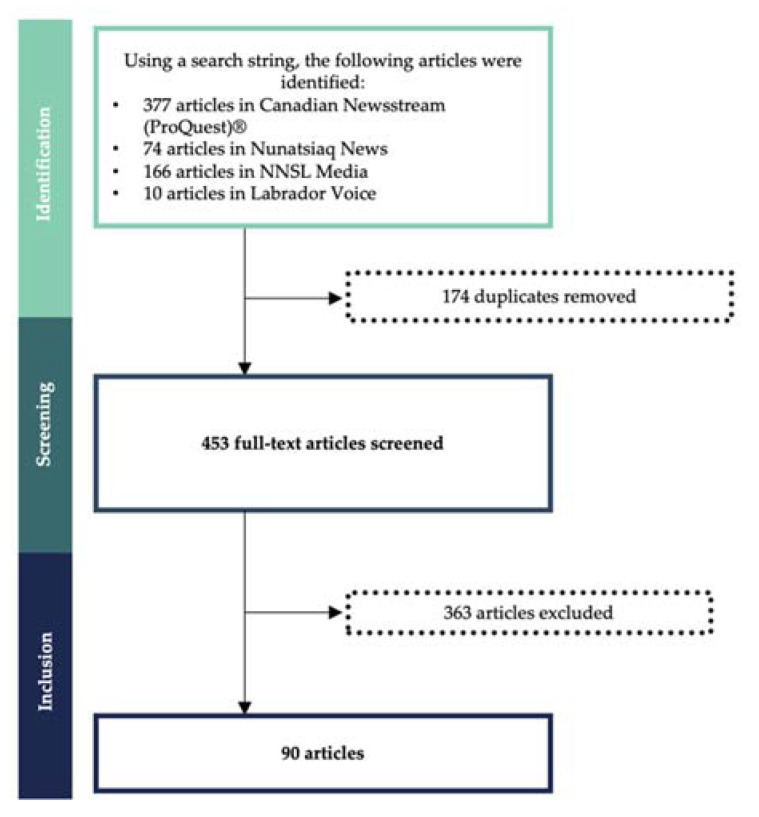
Flowchart of included articles on the mining industry’s initial response to COVID-19 in Inuit Nunangat from 1 January 2020, to 30 June 2020.

The search strategy returned a total of 627 articles, with 453 articles remaining after de-duplication (Figure 2) (de-deplucation involves removing articles that are caught in the search term and show up multiple times in the initial search. Additionally, several media conglomerates such as Postmedia, Saltwire Network and NNSL Media produce the exact same article in many of their numerous newspaper brands they own. For example, Postmedia has over 120 brands it can publish an article under [56]. In these circumstances, only one copy of the article was maintained and recorded). After screening, 90 articles met the inclusion criteria and were analysed. The level of agreement between the two independent reviewers during full-text screening was 98.9%. 

#### 3.1.2. Most Articles Were from Inuit Nunangat-Based Newspapers and Had a Neutral Valence

**Figure 3:**
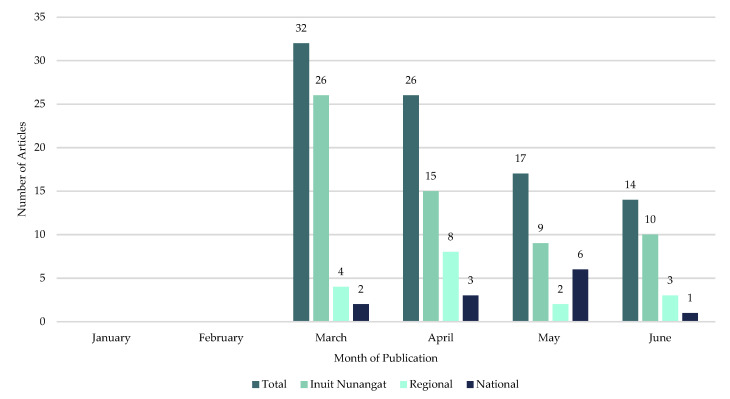
Month of publication, by region, of included articles on the mining industry’s initial response to COVID-19 in Inuit Nunangat from 1 January 2020, to 30 June 2020.

The first article that met the inclusion criteria was published on 11 March 2020. The number of articles peaked in March and decreased monthly thereafter (Figure 3). Month over month, Inuit Nunangat-based newspapers published the most articles included in this study. Except for May 2020, regional newspapers published more articles than national newspapers as national newspapers published the least number of relevant articles for this study.

**Figure 4:**
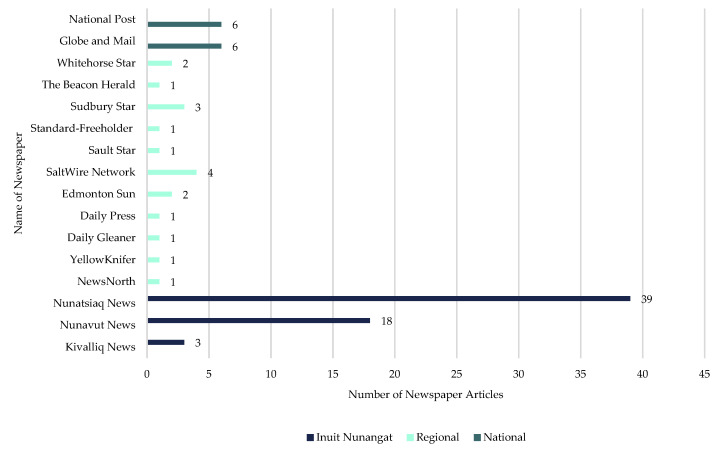
Number of articles, by publisher and region, of included articles on the mining industry’s initial response to COVID-19 in Inuit Nunangat from 1 January 2020, to 30 June 2020.

Inuit Nunangat-based newspapers published 67% (*n* = 60 articles) of all included articles (Figure 4). Regional newspapers located mostly in southern Canada (10 provinces) published 20% (*n* = 18 articles) of the total included articles. National newspapers, namely the National Post and The Globe and Mail, each published six articles, representing 13% (*n* = 6 articles each) of the total included articles.

**Figure 5:**
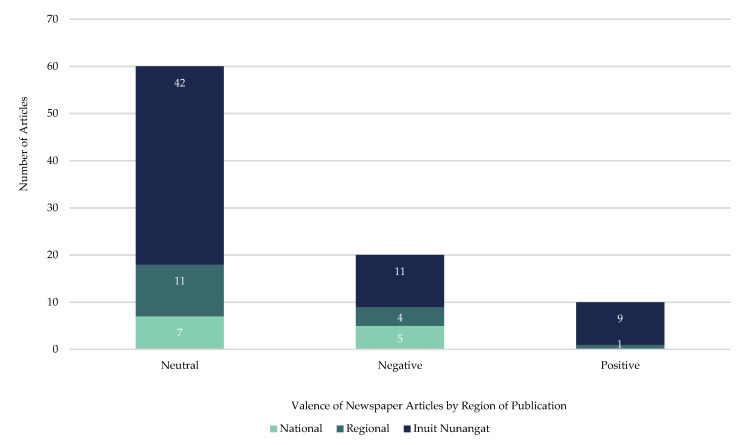
Breakdown of overall valence, by region of publication, of included articles on mining industry’s initial response to COVID-19 in Inuit Nunangat from 1 January 2020, to 30 June 2020.

The majority (67% of total articles/*n* = 60 articles) of articles had a neutral valence (Figure 5). Most often the articles presented both positive and negative statements, which were categorized as having an overall neutral valence. For example, a National Post article from mid-April 2020 outlined Agnico Eagle’s^®^ efforts to resume production with on-site COVID-19 testing, this after a mine road blockade by residents in Rankin Inlet forced the company to act quickly and send home local workers:

*In March, the company scaled back operations at the mine after local residents staged a roadblock to protest the risks posed by flying workers in. Since then, the Nunavut-based workforce is no longer coming to the mine and the workers that are flown in are not interacting with the community. But with no date in sight for when the coronavirus threat will pass, the company is laying the groundwork for an eventual resumption of operations. Instead of waiting for COVID-19 test samples to travel thousands of kilometres to a lab in lower Canada, the company will be able to know within a matter of hours if someone on the site is infected [57].*

In contrast, 22% (*n* = 20 articles) of total articles included an overall negative valence. For example, in mid-March 2020, Baffinland^®^ was in the process of conducting community hearings for their mine expansion proposal and, to prevent the transmission of COVID-19, the regulator decided to change the format from in-person hearings to written submissions and teleconferencing [58]. This decision, and the manner it was reached, was reportedly not well received, particularly by Eric Ootoovak, Chair of the Mittimatalik Hunters and Trappers Organization, who was quoted saying: “The leverage would be in Baffinland’s favour…I feel we are being left out in these important decisions that they’re making without anyone else’s input. It almost is like they’re in favour of the proponent” [58]. Similarly, the articles indicated Agnico Eagle’s^®^ efforts to navigate the regulatory process during the pandemic were reportedly not well received and the Mayor of Rankin Inlet was quoted as saying he did not believe COVID-19 was fully to blame. As the company was seeking approval to drain the contents of a catchment pond containing rain and snow melt water from the mine into a nearby lake, Mayor Harry Towtongie reportedly felt excluded:

*Agnico Eagle has always given us a heads up on what they’re doing, but it happened differently this time and I don’t think that can all be blamed on COVID-19… This action was taken through HTOs (hunters and trappers organizations), KIA (Kivalliq Inuit Association), NTI (Nunavut Tunngavik Inc), the Nunavut Water Board and NIRB (Nunavut Impact Review Board)—it went through all those and the federal minister, while the community, itself, was among the last to hear about it. That just doesn’t sit well with what people are going to think about it, especially our Elders, who are not happy about now having to drink water that has been pumped back into a lake that they’ve been using for so many years [59].*

In total, 11% (*n* = 10 articles) of total articles had an overall positive valence. For example, under the headline “Baffinland lends a hand: mining company donates over $200,000 during COVID crisis [60]”, the story details the monetary contribution to support communities in Nunavut:

*Baffinland Iron Mines has donated more than $200,000 to assist five north Baffin communities during the COVID-19 pandemic. The mining company and its employees put in excess of $115,000 toward food security, working closely with the hamlets in Pond Inlet, Arctic Bay, Clyde River, Iglulik and Sanirajak [60]*.

In another instance, the Member of Parliament for Labrador, Yvonne Jones, gave a positive reaction to Vale’s^®^ handling of the pandemic. She told the *Labrador Voice* that Vale^®^ are “doing all the proper things, everything they said they were doing they’ve done; I haven’t heard a complaint about it at all. They’ve gone way above [61].”

From a framing perspective, 70% (*n* = 63 articles) of total articles had an episodic frame while 30% (*n* = 27 articles) had a thematic frame. For example, in May 2020, an article focused Agnico Eagle’s^®^ plans to return the Nunavut-based workforce and solely focused on this company and their mines [62]. The company’s Nunavut-based workforce were asked to stay home, with pay, as a precautionary health measure. However, another article [63] discussed the plans of several companies to return Nunavut-based workers to the mine sites and this article was classified as thematic. Other thematic frames included topics such as operational impacts to and financial relief for the mining industry in Northern Canada due to COVID-19, on-site testing of employees at various mine sites, wage continuation for Inuit Nunangat-based employees, and continuation of regulatory reviews and public consultations during the pandemic.

#### 3.1.3. Nunavut-Based Mines Mentioned Most Often and Half of All Articles Had In-Depth Discussion on Impact to Operations Due to COVID-19

**Figure 6:**
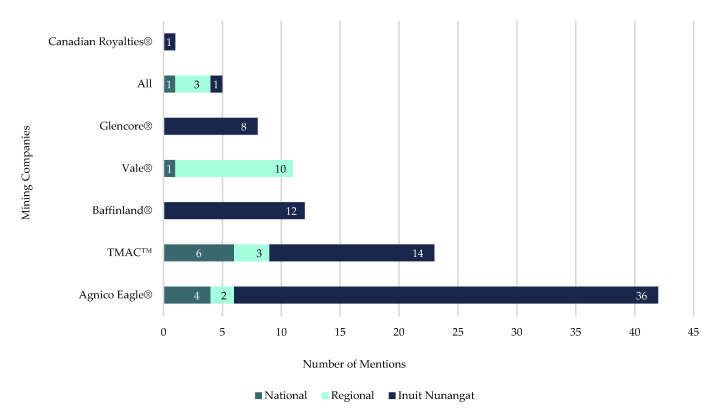
Number of times a mining company was mentioned in articles, by region of newspaper publication, in included articles on mining industry’s initial response to COVID-19 in Inuit Nunangat from 1 January 2020 to 30 June 2020.

Mining companies operating in Inuit Nunangat were mentioned 102 times across 90 articles (Figure 6), with Agnico Eagle^®^ mentioned most often (41% of total mentions; *n* = 42 articles). TMAC™ was mentioned just over half as often (23% of total mentions; *n* = 23 articles), while Baffinland^®^ (12% of total mentions; *n* = 12 articles) and Vale^®^ (11% of total mentions; *n* = 11 articles) combined appeared as much as TMAC™. Glencore^®^ (8% of total mentions; *n* = 8 articles) and Canadian Royalties^®^ (1% of total mentions; *n* = 1 article) were mentioned the least and the Inuit Nunangat mining industry as a whole were mentioned in 5 (5% of total mentions) articles.

Nunavut-based mines represented 75% (*n* = 77 articles) of the mines mentioned, while Nunatsiavut-based and Nunavik-based mines represented 11% (*n* = 11 articles) and 9% (*n* = 9 articles) of the mines mentioned, respectively. There were no operating mines based in Inuvialuit during this study period. In total, 51% (*n* = 46 articles) of articles had an in-depth discussion on the impacts of mining operations due to COVID-19, 23% (*n* = 21 articles) of articles briefly mentioned an impact to mining operations, and 26% (*n* = 23 articles) of articles did not mention any impact to operations.

#### 3.1.4. Half of All Sources Quoted Were Mining Companies and Most Reactions to Industry’s Response Were Negative

**Figure 7:**
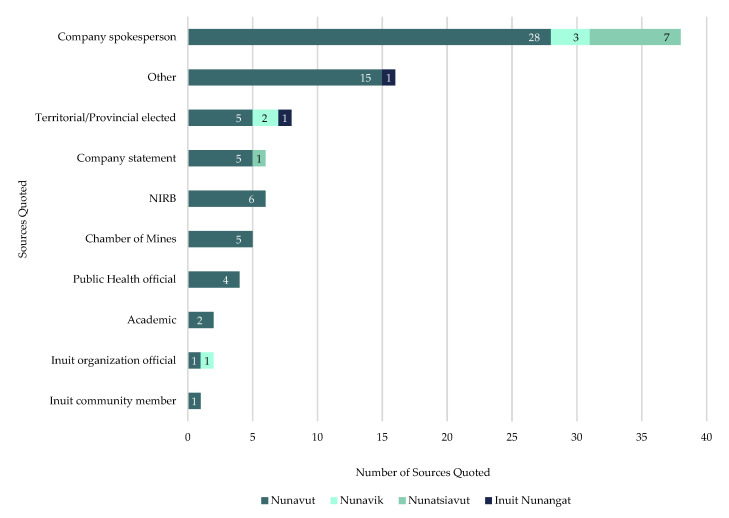
Number of sources quoted, by region, in included articles on mining industry’s initial response to COVID-19 in Inuit Nunangat from 1 January 2020 to 30 June 2020.

A total of 88 sources were quoted (Figure 7) across all included articles, with company spokespersons representing 43% (*n* = 38 of total sources quoted) of all quoted sources. 9% (*n* = 8 of total sources quoted) of sources quoted were provincial or territorial elected officials, while public health officials (5% of sources quoted; *n* = 4 of total sources quoted), Inuit organization officials (2% of sources quoted; *n* = 2 of total sources quoted), academics (2% of sources quoted; *n* = 2 of total sources quoted), and Inuit community members (1% of sources quoted; *n* = 1 of total sources quoted) represented a combined 10% (*n* = 9 of total sources quoted) of total sources quoted. “Other” sources represented 38% (*n* = 33 of total sources quoted) of total sources quoted with company statements (with no spokesperson identified) representing 7% (*n* = 6 of total sources quoted), Northwest Territories and Nunavut Chamber of Mines (Chamber) and the NIRB each representing 6% of total sources quoted (*n* = 5 sources each).

**Figure 8:**
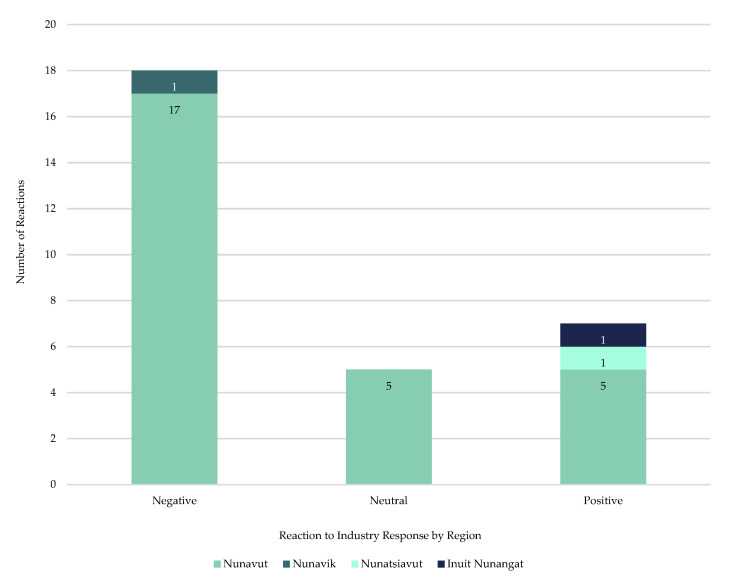
Reactions to Industry response to COVID-19, by region, in included articles on mining industry’s initial response to COVID-19 in Inuit Nunangat from 1 January 2020 to 30 June 2020.

In total, there were 30 reported reactions to the sources quoted regarding the mining industry’s initial response to COVID-19 in Inuit Nunangat (Figure 8). In total, 60% (*n* = 18 of total reactions) reactions were classified as negative, 23% (*n* = 7 of total reactions) were noted as positive, and 17% (*n* = 5 of total reactions) were classified as neutral. Inuit community members provided the most numerous reactions (42%/*n* = 13 of total reactions) and were all classified as negative. The Meliadine mine road blockade by Rankin Inlet residents in March 2020 was noted as an example of a negative reaction to Agnico Eagle’s^®^ initial response to COVID-19, an event *Nunatsiaq News* reported the company’s CEO, Sean Boyd, neglected to mention while discussing pandemic challenges at a June 2020 online Canadian Mining Symposium event: “One challenge that Boyd didn’t mention was how worries among Nunavut residents about the spread of COVID-19 from the company’s southern miners sparked a blockade of the access road from Rankin Inlet to Meliadine in March [64].”

Inuit organization officials’ reactions (17%/*n* = 5 of total reactions) were recorded as negative, with the exception of one noted as neutral. Territorial or provincial elected officials had the most positive reactions (10% of total reactions/*n* = 3 reactions) along with one (3% of total reactions) negative reaction. There were no reported negative reactions from Public Health officials, but they did tally two (7% of total reactions) positive and one (3% of total reactions) neutral reaction. Nunavut’s Chief Public Health Officer, Dr. Michael Patterson, for example, stated neutrally he:

*Expressed great respect for the experts that Agnico Eagle has hired to oversee the mine site laboratory, but he noted that the rapid testing method in question has an error rate of approximately 30 per cent when attempting to determine whether an individual without symptoms might develop the coronavirus [65].*

Despite this, Dr. Patterson stated:

*I have no intention of slowing it down. But I think we have to be very clear about the limitations of a test and the limitations about some of the strategies that have been proposed. Using it to take the place of 14 days of isolation is dangerous [65].*

### 3.2. Reported Themes

Many themes arose in the included articles about COVID-19 and mining operations. These themes are summarized in Table 4 and detailed below. 

#### 3.2.1. COVID-19 Transmitting to Mine Sites and Communities Most Reported Concern

Newspaper articles frequently communicated concern about southern Canada FIFO employees transmitting COVID-19 to a mine site (appeared in 18%/*n* = 16 of total articles) and subsequently transmitting COVID-19 into Inuit communities (appeared in 34%/*n* = 31 of total articles) through a local employee. The Meliadine mine road blockade occurred after Agnico Eagle’s^®^ Vice President of Nunavut Operations told *Nunavut News* on 16 March 2020, they would not be sending Inuit workers home like Baffinland^®^ did, as:

*Inuit employees are integrated and they play a key role, an important role in the operation—we’re talking between 400 and 500 people. All employees and contractors are essential to our operation so this is not an option we’re looking at for now [66].*

Several days later, the company’s CEO reversed the decision and spoke of the added responsibility of operating in Nunavut: “Agnico Eagle has a responsibility to provide a safe workplace and our priority is to protect the health and safety of our employees and the surrounding communities, especially in a context such as Nunavut [67].” Agnico Eagle^®^ then formally announced they would be sending home all Nunavut-based workers, with pay: “This precautionary measure is being implemented in order to eliminate the potential risk of transmission of COVID-19 from a southern worker to a Nunavut worker, with the risk of it moving into the communities” [68]. In response, some involved in the blockade were reportedly pleased with the decision but frustrated with what they had to do:

*We’ve never experienced anything like this before. It’s scary, worrisome and concerning. It’s surreal. This is really happening. Hopefully everyone’s happy with AEM’s announcement, but, at the same time, it’s still scary because these bigwig workers who will still be coming to town might carry the virus. This announcement should have been made two or three weeks ago, not on 19 March. They didn’t seem to take it seriously at all and it shouldn’t have taken a protest to make them do it [69].*

Still, some residents spoke of the challenges of living in the North: “bad enough living up north, we don’t have proper equipment, a building and staff to handle this kind of epidemic. For the safety of our towns, all mines should close too! Baffin and TMAC sent their Inuit home [70].” Another resident reportedly asked:

*If anyone can explain to me how the Agnico Eagle Mine in Rankin Inlet and Baker Lake, Nunavut, is an essential service? They are flying people in and out every day to pull gold out. That gold will still be there tomorrow, next week, next month, even next year. No amount of gold or money would replace a loved one if they pass away from COVID-19 [70]?*

Similar to Nunavut, Nunatsiavut Government President Johannes Lampe gave what was called a blunt assessment of the region: “We are not prepared. We don’t have many of the basic medical things many Canadians take for granted. We don’t have the services we would need if someone became sick. That’s why we are very concerned [17].” Nunatsiavut residents also expressed concern about the history of pandemics in their region:

*I think people here understand for the most part what happened in 1918…We lost a third of our people because of that horrible disease. It’s definitely on everybody’s minds…This virus would be particularly devastating up here, because of the overcrowding situation. There’s a lot of people here with pre-existing health conditions, from TB to diabetes [17].*

For Vale^®^, they told a local newspaper that their decision to enter into care and maintenance in March 2020 was about protecting the region:

*At the rapid pace that the Coronavirus was and continues to spread around the world, we felt it was our responsibility to minimize the potential that it could end up on our doorstep in Northern Labrador, where we have a deep understanding of the realities of the health-care system and the social determinants of health in Nunatsiavut and Innu communities [61].*

Similarly, in Nunavik, Makivik Corporation’s, which represents all Nunavik Inuit, President Charlie Watt was reported to strongly oppose Quebec’s decision to reopen the mining industry in April 2020:

*We are very concerned about the spread of the coronavirus as a result of reopening the mines. We don’t believe the conditions will completely protect the Inuit population from coming in contact with potentially infected people returning to the region [71].*

#### 3.2.2. Wage Protection for Inuit Nunangat-Based Employees

The topic of wage protection appeared in 11% (*n* = 10 of total articles) of total included articles. For Vale^®^, the continuation of wages, albeit at a reduced level with the anticipated support of the Canada Emergency Wage Subsidy (CEWS), was reportedly aiming to offer “both the comfort and security of continued wages for employees during this period of uncertainty and protection and preservation of jobs when operations resume [61].”

After Agnico Eagle^®^ sent all their Nunavut-based employees home, they announced their 400–500 Nunavut employees would continue to receive a wage, again at a reduced rate with the anticipated support of CEWS [62]. Weeks later in April 2020, the company emphasized their rationale for doing so:

*It is our objective to maintain the employment of our Inuit workforce throughout these difficult times as much as possible. The situation will be reevaluated and employees and contractors will be notified when the situation changes. The decision to return our Inuit employees and contractors to work will be made in partnership with the concerned authorities [63].*

For Baffinland^®^, they reportedly shared a similar sentiment, but would discontinue paying on-site premiums:

*Our goal in making this change is to help make our operation sustainable for the duration of the crisis and to avoid layoffs. We continue to evaluate whether any additional measures are required and have committed to employees that we will be as transparent as we can about our decision-making [63].*

For TMAC™, they reportedly were forced to layoffs and felt the CEWS came too late:

*In summary, federal relief programs came too late to influence our decision to slow down operations, and we would not be eligible for this employee-focused measure in any case. TMAC™ and Hope Bay contractors are eligible for other federal relief programs if they are not already eligible for regular EI. We are actively assisting our affected workers apply for federal programs for COVID-19 affected staff [63].*

#### 3.2.3. COVID-19 Precautionary Measures

A total of 26% (*n* = 23 articles) of the included articles reported on specific COVID-19 precautionary measures. Mining companies in Inuit Nunangat reportedly began implementing precautionary COVID-19 prevention and detection measures early in the pandemic. For example, Agnico Eagle^®^ quickly partnered with Dr. Gary Kobinger from the University of Laval’s Department of Microbiology and Infectious Diseases to establish an on-site COVID-19 testing facility:

*We are extremely grateful to Dr. Kobinger and his team for allowing us to be a pilot project for this new rapid COVID-19 testing lab as it provides us with an advanced level of protection for our employees and the communities [67].*

Every operating mine in Inuit Nunangat sent their Inuit Nunangat-based [72,73] workforce home to prevent them from transmitting the virus from the mine site back home to their communities. Mining companies, such as Baffinland^®^, also implemented longer worker rotations to reduce the number of flights, reduced on-site personnel to essential workers only, pre-flight COVID-19 symptom screening, daily sanitation of equipment, and, in general, increased cleaning around the mine site [74].

#### 3.2.4. COVID and Uncertainty about the Future of Inuit Nunangat’s Mining Industry

Uncertainty about the mining industry’s future in Inuit Nunangat was reported in 22% (*n* = 20 articles) of total included articles. The uncertainty was reportedly a significant concern for the Chamber:

*We are not really sure how best to determine the cost of the pandemic so far but it will be significant. With companies having higher operating costs to ensure employee safety and compliance with government and health officer orders, paying salaries of Nunavummiut employees that have returned to their communities, combined with lower production, in some cases, at least, the impact will be significant [72].*

Furthermore, the Chamber believed the initial support programs announced by the Government of Canada were not aligned with the realities of Canada’s Northern mining industry, and they pleaded for assistance: “We urgently request the federal government take the unique circumstances of Canada’s North into account and ensure the companies active in its most important and largest private sector industry are provided the supports they need to survive [75].”

All mines were also reviewing their care and maintenance plans, as in late March 2020, the Government of Quebec ordered businesses shut down in the province, including mines in Nunavik. Vale’s^®^ Voisey’s Bay mine near Nain, Nunatsiavut, announced in early April 2020 they would be extending their 1-month care and maintenance period to at least three months, as they continued to monitor the progress and impacts of the pandemic [61]. However, Quebec mines were re-opened shortly thereafter in April 2020 as they were deemed an essential service by the Government of Quebec [71].

Production reductions at operating mines due to the pandemic, namely due to travel restrictions and inability for mining companies to utilize their Inuit Nunangat-based workforce was reported in 11% (*n* = 10 articles) of total included articles:

*We feel the pain: they are essential to all our operations. But it’s worth it to us to not contribute to any higher risk of infection in the communities. We’re using everything in our power to make sure we’re reducing the risk [74].*

#### 3.2.5. COVID-19 Impacts on Consultation Processes

After the beginning of the pandemic, everyone was faced with the new realities of in-person public consultations, as gatherings were advised against [76]. In total, 17% (*n* = 15 articles) of total articles reported companies such as Baffinland^®^ [77] and Agnico Eagle^®^ [78] were attempting to adapt to the realities of the pandemic and continue with community consultations. For Baffinland^®^, the pandemic arrived when they were conducting community consultations for their phase two mine expansion at Mary River [79] and they reportedly were eager to continue [77]. The NIRB announced in mid-March 2020 because of COVID-19, the expansion hearings, even by teleconference, would be postponed to ensure participants had the opportunity to fully participate [79]. Similarly, Agnico Eagle^®^ filed for an emergency approval in late March 2020 for an emergency discharge of water into a nearby lake. Emergency orders normally do not require public consultation, but the Kivalliq Inuit Association (KIA) argued the company has not demonstrated enough urgency to warrant omitting a public meeting to discuss the issue:

*So far, the information provided by Agnico Eagle^®^ has not convinced KIA that the discharge of saline water will not impact fish and other aquatic life, or that removing all of the water from the containment pond is necessary to avoid an emergency [78]*.

Furthermore, KIA noted, amongst other matters, the pandemic has strained their resources and have made it difficult to consider the company’s emergency request: “This crisis has both limited and stretched KIA’s resources, and has made it particularly difficult to consider Agnico Eagle’s application on short notice [78].” 

#### 3.2.6. Takeover of TMAC™

The takeover bid by Chinese-owned Shandong Gold (SD Gold) for TMAC’s™ Hope Bay gold mine in Nunavut appeared in 11% (*n* = 10 articles) of total included articles. Half (*n* = 6 articles) of the included articles published by national newspapers focused on this issue. The reported interest in the takeover of TMAC™ was mostly in the area of national security and concerns raised by the Canadian government, and others, about foreign acquisitions of Canadian-based companies as the pandemic’s global economic devastation had significantly reduced the value of many publicly traded companies, making them vulnerable for takeover by foreign companies. The acquisition of an Arctic-based mine by a Chinese state-owned company raised specific concerns about sovereignty, given the mine’s proximity to tide water and the Northwest Passage [80]. In TMAC’s™ view, especially in light of the collapse of the economy and commodity prices due to COVID-19, SD Gold had “the financial strength, technical capability and long-term vision to maximize the value of the Hope Bay property [81]” and the impact of an intervention by the Canadian government on the sale of the mine was reported as bleak:

*Without a significant investor like SD Gold, the mine’s current owners stated they would likely shut down operations, leaving Nunavut workers without a job, suppliers without a customer and the Kitikmeot Inuit Association without the benefits associated with the gold mine [81].*

## 4. Discussion

The objective of this study was to characterize the nature, range, and extent of national and regional newspaper coverage of the mining industry’s initial response to COVID-19 in Inuit Nunangat between 1 January and 30 June 2020. This review suggests Inuit-Nunangat-based newspaper coverage was instrumental in shaping the narrative of the mining industry’s initial response to COVID-19. If readers were to solely rely on national or southern Canadian regional newspaper coverage, based on these results, it would likely be narrowly limited without a local lens to fully provide relevant context and reaction from Inuit Nunangat-based residents and officials. This finding is consistent with another newspaper study focusing on northern Canada which found that when reviewing general issues (i.e., beyond COVID-19) impacting the North, local media such as *NNSL* and *Nunatsiaq News* provided the majority of relevant articles when national and southern Canadian regional newspapers were included in the search [27]. Indeed, Inuit Nunangat-based media provided more in-depth and relevant coverage and set the agenda for the type of coverage provided. For example, *Nunatsiaq News* reporter Jane George was profiled in an academic journal and stated her organization’s influence in the region, as readers are known to read every word [25]. George, when speaking about climate change in the North for example, also appeared to understand with her influence comes a heightened responsibility to unravel complex topics to make them interesting and understandable for her target audience in the North [25]. In contrast to locally based reporting, research focusing on media coverage on Arctic Canada, discussed how national newspapers report on the region from afar or “parachute” reporters into the region for a brief period of time [31]. National newspaper coverage likely does not have a local audience in mind but parachute coverage can be viewed as exploitive in nature, as they arrive, take information for their own purposes, and then depart until the next news cycle requires their attention [31]. For Inuit Nunangat, local newspapers such as *Nunatsiaq News* have historically been a significant supplier of local news, often times being the only source of local news for some Inuit communities [25]. 

Most of the included articles for this review were reported by Inuit Nunangat-based newspapers and the majority focused on Nunavut-based mines. A likely explanation is both *Nunatsiaq News* and Northern News Services Limited’s (NNSL) *Nunavut News* focus heavily on Nunavut and four of the seven operating mines in Inuit Nunangat are in Nunavut. The earliest dated article that met the inclusion criteria appeared on 11 March 2020, when TMAC™ outlined their plans to update their infectious disease plan. At the time, *Nunatsiaq News* reportedly attempted to contact Baffinland^®^ and Agnico Eagle^®^ representatives to obtain their infectious disease plans, but a response was not provided prior to the journalist’s deadline [82]. In the same article, the newspaper coverage discussed the planned Nunavut Mining Symposium due to take place between 30 March and 2 April 2020 and emphasized there were no recommendations to cancel the event. Within ten days of this article publishing, all mines had their Inuit Nunangat-based workforce returned home and travel restrictions implemented for their southern Canada-based workforce. The newspaper coverage of this significant escalation period would be scarce if not for Inuit Nunangat-based newspapers such as *Nunatsiaq News* and NNSL Media’s several weekly newspapers. The in-depth coverage was mostly missing on a Canada-wide basis with national newspapers focusing mostly on the takeover of TMAC™ by Chinese state-owned SD Gold [83], with the focus on national security [84] and not necessarily the health and well-being of mine employees, their families and communities. This low amount of coverage by mainstream media is consistent with other reports on media coverage of Indigenous (Some citations make reference for “Aboriginal peoples.” For the purposes of this study, unless a direct quote, we utilize “Indigenous” when collectively referring to First Nations, Inuit and/or Metis Peoples in Canada)-related matters. For example, a report on Indigenous-related news stories in Ontario between 2010 and 2013, indicated only 0.15–0.46% of total annual news stories in that province focused on Indigenous peoples, cultures and/or issues [29]. 

Additionally, newspapers in the North were sharing the perspectives of mining companies more than other newspapers were. Half of the sources quoted were mining company spokespersons (43%) or company statements (7%). Acknowledging this study actively sought out the mining industry’s response to COVID-19, half of sources quoted representing mining companies is understandable. However, it also demonstrates newspapers in the North understand how important mining is to the region and likely understood it would be of great interest to readers. However, it was noteworthy that of the 44 times a company spokesperson or statement was quoted, 70% of the time there were no reported reactions to the company’s information. More often than not, mining companies were able to get their messages into newspapers without any Inuit or local response to their statement or actions provided, which could have led to mining companies controlling the narrative of how their response to COVID-19 was actually going. Other research on COVID-19 media reporting also found the reporting to be mostly descriptive and uncritical [23]. A possible explanation for this would be the rapid escalation and significant amount of pandemic-related information becoming available, and a competition amongst media to immediately report the latest available information as breaking news [85] as the public was pressuring to get COVID-19 facts immediately [23]. 

There were 31 reactions to the mining industry’s initial response, with 43% (*n* = 13 of total reactions) from Inuit community members, all noted as negative reactions. One important point to consider is Inuit mining company employees often are not quoted or represented in the media, as it is likely they have social media [86] and media policies in place that would prevent them from doing so. With mining operations providing some of the highest paying jobs [22] in Inuit Nunangat, employees and their family members could possibly fear retribution for any public comments, whether positive or negative, and thus remain silent. It is not evident any of the quoted community members are employees or family members of employees, but it appears, based on the reporting, the Inuit community members felt the negative health impacts associated with the pandemic outweighed any potential negative socioeconomic impacts as many of them wanted the FIFO mines shut down altogether [70]. 

Inuit are not only keenly aware of the impacts of the FIFO nature of the mining industry, but life in general, as they often have to fly to southern Canada for medical care not readily available in their communities [24]. With no roads between communities, or between their region and southern Canada, food and supplies need to be flown in as well. FIFO is often part of the lives of many Inuit [24] and in the context of a highly contagious virus, Inuit community members’ calls to stop the mass influx of southern Canadian mining workers to their region is quite understandable. In the case of Nunatsiavut, after having commemorated the 100 year anniversary of the 1918 Spanish Flu pandemic [18], they were aware that their population avoided the first of three waves of that pandemic as they remained isolated and did not have contact with the southern population [24], a strategy many Inuit were attempting to replicate as much as possible. Indeed, health risks associated with FIFO mining operations were a concern prior to COVID-19 and the pandemic has only exacerbated the health risks as FIFO workers are potential vectors through which COVID-19 could be transmitted into Inuit communities [10]. Similarly in other jurisdictions in the North, Indigenous leaders were concerned with the FIFO mining workers and the potential health impacts on their communities. In the Yukon, for example, Indigenous leaders balked at conflicting messaging from the Premier as he declared a state of emergency to stop the spread of COVID-19 while allowing mining to continue [10]. To a larger extent, some have suggested the pandemic will impact the political struggles between Indigenous communities and mining companies over the impacts and benefits of mining operations, leading to demands that Indigenous communities sacrifice their health, environment and political rights in the name of profits and a sustainable business model [10]. However, it was also suggested that the “determined resistance from Indigenous communities” is cause for optimism as well [10].

In terms of valence, the positive stories mostly reflected the decision of mining companies to send Inuit Nunangat-based workers home with pay, along with stories discussing corporate donations to support communities through the pandemic. However, the most pressing issue reported at the onset of the pandemic was COVID-19 transmitting to the mine site and subsequently into communities in Inuit Nunangat [68,87,88,89]. For many Inuit, the mining industry represents one of a few opportunities to remain in their home communities while still earning a high salary [22]. For companies, there appears to be some recognition of this added responsibility and power they hold in Inuit Nunangat, as during the initial stage of the pandemic, mining companies reportedly were concerned with COVID-19 entering their mine sites and ultimately the surrounding Inuit communities [68,88,89]. One could argue it was strictly out of a sense of liability to protect shareholders. However, sending Inuit employees home with pay suggests two things: companies were mostly understanding of their social license to operate and they had to take whatever short-term financial losses necessary to ensure the operation had a future. Companies such as Baffinland^®^, Agnico Eagle^®^, and Vale^®^ sent employees with varying wage coverages with job security guarantees. There is no evidence to suggest mining companies across Inuit-Nunangat cooperated, but there is evidence to suggest these companies understood, or quickly learned during the pandemic because of public pressure, the social responsibilities expected of them in working in Inuit Nunangat [68,88,89]. Vale^®^, the Brazilian headquartered global mining company, had mines in Manitoba (Thompson), Ontario (Sudbury region), and Newfoundland and Labrador (Voisey’s Bay, near Nain, Nunatsiavut). While Vale^®^ kept their Manitoba and Ontario mines open, they put the Voisey’s Bay mine into care and maintenance and sent most of their employees home with reduced salaries on 16 March 2020. The company stated that with more than 50 per cent of their workforce being Indigenous (Innu and Inuit), and acknowledging a bad history with pandemics in the region, they did not want to accept the risk of a worker bringing the virus to the mine site and then back into a remote northern community, where healthcare options are limited [89]. 

However, not all companies immediately made well-received decisions at the commencement of the pandemic and their initial response was likely influenced by local newspaper coverage. For example, on 16 March 2020, Agnico Eagle’s^®^ Vice-President of Nunavut Operations told *Nunavut News* Inuit employees were integral to their mining operations and sending them home was not an option [66]. Three days later, the company’s CEO informed the same newspaper they decided to send all their Nunavut-based workforce home with reduced salaries, as they valued their relationship with the people of Nunavut and were committed to doing what was best for the health and safety of their employees and the communities they live in [68]. The stark difference in response on 16 March compared to 19 March suggests the 16 March comments by the company official were not well received and it required an escalation to the CEO level.

Specifically relating to the Inuit-Nunangat based employees, whether companies knew it or not, sending employees home with pay and job security was not just a business decision, but a health-based decision as well [13]. A number of Inuit depend on mining jobs for remaining in their home community, and as one Inuit mine employee suggested in an academic study, she would not be able to stay in her Nunavut community if the mine closed and predicted the impact would be severe, leading to her and other employees to leave their home community [22]. By maintaining their jobs with guaranteed but reduced salaries, the impact on their livelihoods, social status, mental wellness, and personal safety and security was likely lessened. Additionally, a sudden panic by mine employees to leave their communities to secure employment elsewhere was likely avoided by the continuation of wages by several of the mining companies. Indirectly, housing and food security were likely not significantly impacted given the continued of employment and income [13]. However, there were potential negative impacts of workers remaining home rather than on their usual rotational work schedule. Confined to the household and occupying space in what could be an overcrowded home and an increase in the costs of groceries, electricity and gasoline due to the employee being home, rather than at the mine site and offsetting those normal monthly costs [22]. A loss of routine, potential loss of purpose, potential shame of southern workers who still have to fly to the mine site and work to earn their wages, and increase in substance misuse are other potential impacts [22]. It is important to note there were no articles reflecting any disgruntlement from southern Canada workers about the decision to send Inuit Nunangat-based workers home with pay. However, there were also potential positive impacts for Inuit employees: more time on the land, more opportunity to hunt and fish, more time with family [22], and potential alleviated stress by not having to interact with southern Canada workers. From the reporting, it appears sending the Inuit-Nunangat based workers home with pay was implemented with good intent [68,88,89] and there was likely insufficient time for full analysis of the unintended consequences of such action. The reported immediate urgency was to stop the virus from transmitting into Inuit communities and action needed to be taken to do so with the least impact possible. Despite the actions taken by the mining industry, whether voluntary or imposed, to protect the health of Inuit Nunangat-based employees and communities, there was a scant amount of reporting on the potential health impacts of sending Inuit employees home for several months. This is significant, as there are potential serious direct and indirect health consequences from COVID-19. In Nunavut, COVID-19 threatens the territory’s entire population, as the combination of already-present underlying health conditions and lack of access to health-sustaining resources, and other complex social determinants of health (the Inuit Public Health Task Force (IPHTF) focused on the following areas in determining the Inuit-specific social determinants of health: quality of early childhood development, culture and language, livelihoods, income distribution, housing, personal safety and security, education, food security, availability of health services, mental wellness, and the environment [13]) could lead to far more serious impacts to a population compared to southern Canada [70]. Additionally, the pandemic threatened to shut down an industry representing approximately 38% of the territory’s Gross Domestic Product (GDP) [12] [72].

For Nunatsiavut, the situation is similar, as leaders and residents were intimately aware of the potential impacts of a pandemic. Their understanding of, and attention given to, the social determinants of health distinctly concerned local leadership, but also ensured they were prepared to implement the necessary preventative measures at their disposal to prevent the virus from reaching the communities, as it was reported it would be difficult to manage the spread of the virus if it did reach the region [17]. The identified lack of in-community medical personnel and equipment, the overcrowding in housing, pre-existing conditions such as diabetes and tuberculosis, and the FIFO nature of receiving basic and advanced medical care in Nunatsiavut makes them extremely vulnerable in a pandemic [17]. One resident specifically commented on the region’s understanding of the 1918 Spanish Flu and the impact it had on Inuit in Labrador [17]. 

While newspaper coverage originally focused on the immediate actions of mining companies in response to COVID-19, coverage began shifting towards actions of mining companies to adapt to the new reality of conducting business in Inuit Nunangat during a pandemic. Despite a significant amount of uncertainty of how the pandemic would impact the future of the industry, mining companies were eager to adapt to the new realities of conducting community consultations while Inuit organizations struggled to keep up with many competing demands. Half of all negative reactions to the mining industry’s initial response to COVID-19 were related to companies wanting to conduct consultations while the pandemic was in the early stages of impacting Canada. Baffinland^®^ attempted to conduct public hearings on its mine expansion [77], Agnico Eagle^®^ applied for an emergency water release and wanted to build a water pipeline [90], and Glencore^®^ resumed operations in Nunavik with the approval of the Quebec government, but Nunavik Inuit were not consulted and felt the move to resume operations was premature [71]. 

For Inuit and Inuit organizations, community consultations are integral to the success of mining developments in Inuit Nunangat [91]. Inuit must be meaningfully consulted on matters of importance, including significant matters such as a proposed mine expansion [79], emergency water release [78] and construction of a water pipeline [91]. However, the same Inuit organizations who would be responsible for contributing to this consultation in a meaningful way were also responsible for the immediate and unprecedented pandemic response [76]. While there was recognition of the importance of the mining industry’s requests, the health and safety of Inuit and their communities was reported as a much higher priority, despite the mining industry’s portrayal of urgency [78]. It also is likely a blemish on the record of support the mining companies provided, as they demonstrated an understanding, either initially amongst themselves or informed by the actions of communities, of the severity of the pandemic and the impact on the region, but reportedly then attempted to carry on with a business-as-usual type of approach. 

Further, the shifts in mining operations during COVID-19 are not just Inuit Nunangat issues, they are Canadian issues. Mines in Inuit Nunangat provide critical jobs for many southern Canadian residents who rely on the FIFO employment opportunities, along with providing employment for all corporate head offices, often located in southern Canada. For example, in March 2020, Baffinland^®^ ordered approximately 300 Nunavut-based employees home but still had approximately 650 workers from southern Canada employed at the mine site [74]. The materials mined are necessary for products we use in everyday life, such as gold—while mostly utilized in jewelry [92], it is also utilized in the medical field [93]. Additionally, Inuit Nunangat mines supply materials required for the shift to a greener economy, as nickel, for example, is required for electric car batteries [94]. However, as a resident in Nunavut reportedly stated, the minerals were still available for future mining and they felt the mines should have closed down in the interest of health and safety [70]. 

From the included articles, all mining companies reported being focused on implementing measures to continuing operating, as shutting down to wait out the pandemic was not reported as a financially feasible option for them. Despite the magnitude of what was at stake, the national newspapers were limited in their reporting on the matter. Other literature suggests despite matters significantly impacting the Arctic, if it is not impacting the day-to-day lives of southern Canadians, it may contribute to a lack of national and regional media coverage [27].

Changing how reporting is conducted in Inuit Nunangat and how non-Indigenous Canadians view Inuit-Nunangat or other Indigenous issues are supported by the 2015 Truth and Reconciliation Report, whereby the Truth and Reconciliation Commission included the following Call to Action:

*We call upon Canadian journalism programs and media schools to require education for all students on the history of Indigenous peoples, including the history and legacy of residential schools, the United Nations Declaration on the Rights of Indigenous Peoples, Treaties and Indigenous rights, Indigenous law, and Indigenous–Crown relations [30]*.

This call to action suggests the lack of national news coverage is deeply rooted and systemic, and requires a long-term approach to educate the future generations of journalists on the responsibility they hold in reporting the stories of Indigenous Peoples across Canada, a responsibility noted by ITK President Natan Obed directly to media while standing next to Prime Minister Trudeau in 2019 [28]. Pertaining to Inuit Nunangat, while financial constraints may be a noted reason national newspapers provide for not having Inuit Nunangat-based journalist [31], this type of training could be helpful to prevent the current exploitive nature of parachute journalism and reporting from afar. In another newspaper study on the North, no southern regional or national paper quoted members of the Indigenous general population and a potential heightened awareness of this type of exploitive relationship by Indigenous Peoples could be an explanation why [27]. 

While this study was systematic in nature, there are limitations to note. Inuit Nunangat-based newspapers were not included in the ProQuest^®^ database, nor was the regional newspaper covering Nunatsiavut. Search engines on Inuit Nunangat-based newspaper websites did not allow for the detailed search string, and simple searches were conducted utilizing two terms (i.e., “COVID” and “mine”). Therefore, there is potential not all relevant newspaper articles were captured. Nunatsiavut does not have a Nunatsiavut-based newspaper, which likely led to the lower number of articles discussing the region. Other forms of media were not captured, and we acknowledge the potential influence of radio, television, online media outlets and social media in Inuit Nunangat, all of which were excluded from this study.

## 5. Conclusions

Despite often providing mining companies with unchallenged messaging in their reporting, Inuit Nunangat-based newspapers played a critical role in shaping the narrative and initial response of mining companies to the COVID-19 pandemic. During the study period, national newspaper coverage of COVID-19 and mining in Inuit Nunangat mainly focused on the takeover of TMAC™, while Nunatsiaq News and NNSL’s several weekly newspapers focused mainly on providing relevant and timely COVID-19-related information for the people who call Inuit Nunangat home. Without Inuit Nunangat-based newspapers providing in-depth and relevant coverage of the initial response, the reporting would have been left to national and southern regional newspapers, which was less in-depth, less frequent, and less relevant. At the end of the study period, newspapers covered the uncertainty about the future of the mining industry and how the pandemic will impact Inuit Nunangat communities. 

## Figures and Tables

**Table 1:** Terms used to search ProQuest^®^ databases and local newspaper outlets (i.e., NNSL media, Nunatsiaq News and Labrador Voice) to identify newspaper articles on COVID-19 and mining in Inuit Nunangat between 1 January 2020, and 30 June 2020.

Component	Search Terms
ProQuest^®^ Database Search	
Location	(“Northern Canada” OR “Canadian North” OR “Canada’s North” OR circumpolar OR Nunangat OR Inuit OR Inuvialuit OR Nunatsiavut OR Nunavik OR Nunavut OR Nunavummiut OR “Northwest Territories” OR NWT OR Labrador OR “Northern Quebec” OR arctic OR sub-arctic OR subarctic OR ITK)
	AND
COVID-19	(COVID-19 OR COVID19 OR COVID OR coronavirus OR pandemic OR quarantine OR isolation OR virus OR health)
	AND
Mining	(mining OR mine OR “resource extraction” OR IBA OR “impacts and benefits” OR agreement OR consult OR “Voisey’s Bay” OR Vale OR Glencore OR Raglan OR “Agnico Eagle” OR Meadowbank OR Meliadine OR Baffinland OR “Mary River” OR TMAC OR “Hope Bay” OR “Canadian Royalties” OR “Deception Bay”)
NNSL Media, Nunatsiaq News and Labrador Voice Search	
COVID-19	COVID
	AND
Mining	mine

**Table 2:** Inclusion and exclusion criteria used to identify relevant articles on mining industry’s initial response to COVID-19 in Inuit Nunangat from 1 January 2020, to 30 June 2020.

Inclusion Criteria	Exclusion Criteria
Article mentioned an operational mine which was physically located in one of the four settled Inuit land claim regions in Canada (Inuit Nunangat): Inuvialuit, Nunavik, Nunavut, and/or Nunatsiavut. Only mining operations in one (or more) of these four regions were included.	Article mentioned a non-operational mine, or only mentioned an operational mine outside of the four regions.
Article mentioned the COVID-19 pandemic in relation to the operations of the mine.	Article did not mention the COVID-19 pandemic in relation to the operations of the mine.
Article was published on or after 1 January 2020, and on or before 30 June 2020.	Article was published before 1 January 2020, or after 30 June 2020.
Article was published in English or French.	Article was published in a language other than English or French.

**Table 3:** Data extraction form utilized for deductive qualitative analysis.

	Category	Options	Notes
Articles Attributes	Name of Newspaper	Open text field	
	Date	Open text field	
	Author	Journalist Opinion column Other	
	Sources Quoted	Academic Company spokesperson Inuit employee Inuit community member Inuit organization official Territorial or provincial elected official Public health official Other (specify) Not applicable	Sources quoted regarding mining industry’s response to COVID-19.
	Frame type	Episodic Thematic	If article only discussed one company, it was classified as episodic. If article discussed more than one company, or the mining industry as a whole, it was classified as thematic.
	Valence	Negative Neutral Positive	Valence regarding the mining companies’ initial response to COVID-19 is assessed. If article contained both positive and negative statements, it was classified as neutral.
Mine-Related Data	Location of Mine	Nunavut Nunavik Nunatsiavut Inuit Nunangat	
	Mining Company	Agnico Eagle^®^ Baffinland^®^ Canadian Royalties^®^ Glencore^®^ TMAC™ Vale^®^ All	
COVID-19	Issues	Case on mine site Case in Inuit community Underlying health conditions Access to medical care Future of employment Wage protection Second wave Other (specify)	
	Reaction to industry response to COVID-19?	Yes No	Noted reactions to the sources quoted.
	If yes, whose reactions were reported?	Academic Company spokesperson Inuit employee Inuit community member Inuit organization official Territorial or provincial-elected official Public health official Other (Specify)	Classification of individual providing a reaction to mining industry’s response to COVID-19.
	If yes, how would you describe those reactions?	Negative Neutral Positive	Overall reaction based on quote or photo provided. If quote contained both positive and negative statements, it was classified as neutral.
	To what extent does the article discuss an impact to mining operations due to COVID-19?	Not at all Briefly mentioned In-depth discussion	If just a sentence, it was classified as briefly mentioned. If a paragraph or more, it was considered an in-depth discussion.

**Table 4:** Summary of deductive and inductive themes, by number of appearances, regarding COVID-19 and mining operations.

**Deductive Themes**	**Appearance in Overall Articles (%/n) ^1^**
COVID-19 transmitting to Inuit communities	34%/31
COVID-19 transmitting to a mine site	18%/16
Wage protection for Inuit Nunangat-based employees	11%/10
**Inductive Themes**	
COVID-19 precautionary measures	26%/23
Uncertainty about future of mining industry in Inuit Nunangat	22%/20
Impacts on consultation processes	17%/15
Takeover of TMAC™	11%/10

^1^ If an issue/theme appeared more than once in an article (i.e., two different companies discussing wage protection) it was counted as one appearance. Percentages calculated based on total number of included articles (90 articles).

## Data Availability

Data are available upon request from primary author.
